# Isolated lateral extra-articular tenodesis enhance better rotatory knee joint stability post-primary ACL repair: Four cases report and literature review

**DOI:** 10.1016/j.ijscr.2021.106167

**Published:** 2021-07-02

**Authors:** Romy Deviandri, H.C. van der Veen

**Affiliations:** a Department of Orthopedics, University Medical Center Groningen, University of Groningen, Groningen, the Netherlands; b Department of Physiology- Faculty of Medicine, Universitas Riau, Pekanbaru, Indonesia; c Division of Orthopedics- Sports Injury, Arifin Achmad Hospital, Pekanbaru, Indonesia

**Keywords:** ALL, Modified Lemaire, ACL reconstruction, Rotatory instability, Lateral tenodesis, Case report

## Abstract

**Introduction and importance:**

Residual rotatory instability has been reported to occur after primary anterior cruciate ligament reconstruction. The anterolateral ligament complex of the knee has gained attention for its role in rotational instability of the knee, especially in association with anterior cruciate ligament injuries. The role of an isolated lateral extra-articular tenodesis procedure among those patients presenting with residual rotatory instability after primary anterior cruciate ligament reconstruction has not been reported on.

**Case presentation:**

Four patients (Tegner level 4) presenting with residual rotatory instability after primary anterior cruciate ligament reconstruction without signs of graft failure, underwent an isolated lateral extra-articular tenodesis with modified Lemaire procedure. Pre- and postoperative outcome scores were assessed. At one-year follow-up, all patients reported functional knee stability. Pivot shift tests were negative and postoperative Lysholm scores were increased with a mean of 19.75 points. Tegner scores equaled the preinjury level.

**Clinical discussion:**

This case report showed that our four patients where successfully treated with an isolated secondary modified Lemaire procedure for residual anterolateral rotatory instability after primary anterior cruciate ligament reconstruction.

**Conclusion:**

An isolated secondary lateral extra-articular tenodesis procedure can be a valuable treatment option for moderate active patients with residual rotatory instability after a primary anterior cruciate ligament reconstruction without signs of graft failure.

## Highlights

• Residual rotatory instability has been reported to occur after primary ACLR
• Adding LET improve patient outcome without the need for ACL revision in patients with residual rotatory instability after ACLR
• This procedure obligatory was followed by intense postoperative rehabilitation

## 1 Introduction

Anterior cruciate ligament reconstruction (ACLR) is an operation frequently performed by orthopaedic surgeons. It is estimated that approximately 400.000–500.000 cases of ACLR are carried out each year in the United States (US) based on implant usage, a number expected to increase further as a result of increased participation in athletic activities by adolescents and young adults [1]. Although ACLRs are routinely performed in an attempt to restore native knee kinematics following an ACL injury, up to 25% of the patients continue to experience persistent anterolateral rotatory laxity post-surgery as assessed by the pivot-shift test [2]. The underlying etiology for this laxity is multi-factorial, including technical errors, biological failure, traumatic injuries, bone morphology and damage to structures comprising the anterolateral complex (ALC) of the knee [3].

The ACL is the primary restraint to anterior tibial translation. It has an oblique orientation close to the center of rotation of the knee, so its lever arm to control rotation is small and so an isolated intra articular ACLR may be relatively ineffective for controlling internal rotation. Not all patients treated with an isolated intraarticular ACLR are rotationally stable, with some having a residual pivot shift, and that might be related to damage to extra articular soft tissue structures [4].

In 1879, The French surgeon Paul Segond described a “pearly, resistant, fibrous band inserting on the anterolateral aspect of the proximal tibia” while describing the eponymous fracture that is now considered pathognomonic of an ACL injury. Hughston and colleagues identified the middle third of the lateral capsular ligament to be technically strong, attached proximally to the lateral epicondyle and distally to the tibial joint margin. In cases with anterolateral rotational instability, they identified it to be torn in 5 acute clinical cases (4 of whom had an ACL injury) and lax in 20 chronic cases (15 with an ACL injury). Recent MRI studies also documented anterolateral ligament (ALL) injuries in 46% to 79% ACL injuries [5]. Experimental studies indicate that the ALL complex contributes to stability against internal rotation particularly in flexion angles beyond 30 degree, reaching peak contribution between 60 and 75 degree of flexion, with significant effect continuing beyond that [6]. It has therefore been suggested that when high grades of pivot shift are detected preoperatively, an unrecognized injury to the ALL complex should be considered and isolated ACLR may fail to restore normal knee stability [7].

In general, most surgeons would treat persistent instability after ACLR with revision surgery, which is a technically challenging procedure. The outcome depends on the surgeon's ability to correctly identify and treat the possible causes of failure of the primary operation [5]. Although various studies demonstrated that adding a lateral extra-articular tenodesis (LET) to an ACLR improved knee stability, decreased rotational knee laxity and decreased the rate of ACL failure [8], the role of an isolated LET among those patients presenting with residual rotatory instability after primary ACLR has not been reported on.

The purpose of this report is to illustrate the role of an isolated LET for patients presenting with residual rotatory instability after primary ACLR. This study is reported in line with the SCARE 2020 guidelines [9].

## 2 Cases report

In 2019, four consecutive patients were identified with residual rotatory instability presenting more than one year after primary ACLR (Table 1). All surgeries were performed by second author (HvdV) as a knee-orthopaedic surgeon in our hospital. All patients gave consent for this treatment.

**Table 1:** Patient characteristics.

	Patient 1	Patient 2	Patient 3	Patient 4
Age (years)	30s	30s	20s	20s
Gender (M/F)	F	F	F	F
Time of ACLR	2011	2007	2014	2018
ACLR Technique	Transtibial (TransFix)	Transtibial (Endobutton)	Anteromedial portal (Endobutton)	Anteromedial portal (all-inside)
Graft type	Hamstring	Hamstring	Hamstring	Hamstring
Graft Intact	Yes	Yes	Yes	Yes
Graft Position	Steep orientation	Steep orientation	Anatomic	Anatomic

Abbreviation: M, male; F, female; ACLR, anterior cruciate ligament reconstruction.

**Fig. 1:**
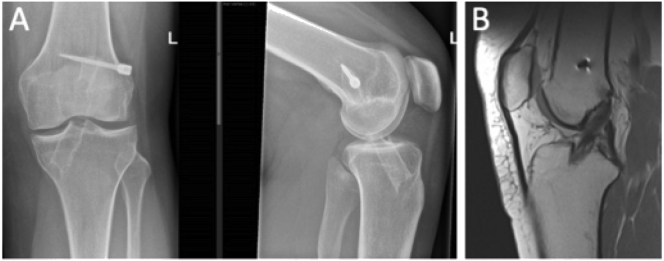
A. Plain X-rays AP and lateral left knee (patient 1). Steep orientation of the graft. B. Sagittal MRI showing intact ACL graft (patient 1).

The first patient was female in her 30s, who underwent a transtibial ACLR in 2011. At presentation in 2019, she complained of instability since one year, which was probably developed during fitness exercises and jumping, without history of a clear distortion. Testing her knee showed Lachman 1+, and Pivot Shift grade 2 with a recognizable feeling. Physiotherapy had not been effective so far. Plain X-Ray and MRI showed an intact ACL graft with steep orientation of the graft (Fig. 1).

**Fig. 2:**
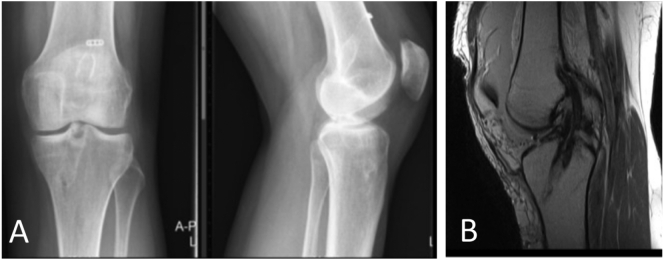
A. Plain X-rays AP and lateral left knee (patient 2). Posterior position of the tibial tunnel, steep orientation of the graft. B. Sagittal MRI image of an intact ACL graft (patient 2).

The second patient was a female in her 30s, who underwent a transtibial ACLR in 2007. She presented in 2019 after a slight knee distortion with increased feelings of instability afterwards. It never had felt fully stable, but now it was influencing her daily activities. Knee stability tests showed Lachman 2+, and Pivot Shift grade 2, with a recognizable feeling of instability. Physiotherapy was not effective. Plain X-Ray and MRI showed an intact ACL graft with steep orientation of the graft and a too posterior position of the tibial tunnel (Fig. 2).

**Fig. 3:**
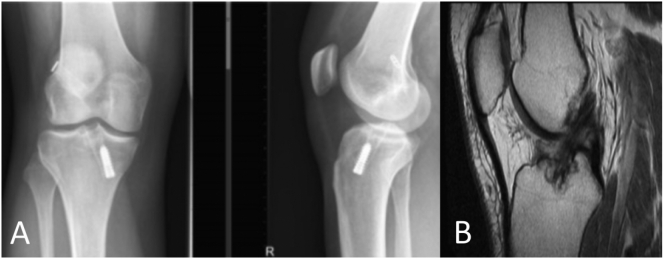
A. Plain X-rays AP and lateral right knee (patient 3). Adequate tunnel positioning. B. Sagittal MRI image of an intact ACL graft (patient 3).

Our third patient was a female in her 20s, who underwent an ACLR with endobutton anteromedial portal technique in 2014. Her knee was more stable since reconstruction, but she still complained of giving way during moderate exercises, with Lachman 1+ and Pivot Shift grade 2. Physiotherapy was not effective. Plain X-Ray and MRI showed an intact ACL graft with adequate tunnel positioning (Fig. 3).

**Fig. 4:**
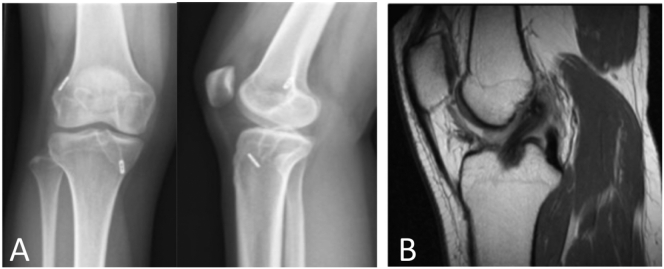
A. Plain X-rays AP and lateral right knee (patient 4). Adequate tunnel positioning. B. Sagittal MRI image of an intact ACL graft (patient 4).

The fourth patient was a female in her 20s, who underwent an ACLR (all-inside technique) in 2018. After one year she still experienced giving way during moderate exercise, with Lachman 1+, and Pivot Shift grade 2. Physiotherapy was not effective. Plain X-Ray and MRI showed an intact ACL graft with adequate tunnel positioning (Fig. 4).

**Fig. 5:**
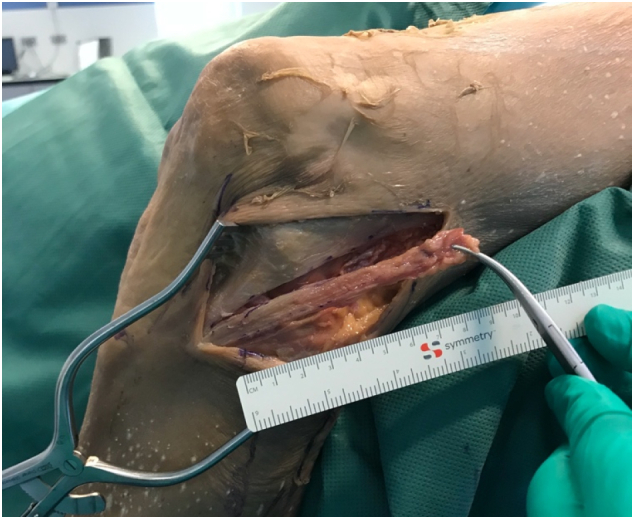
The iliotibial band was exposed and a 9 by 1 cm strip was excised from the middle part of the iliotibial band, leaving its distal end attached to Gerdy's tubercle.

All four patients underwent an isolated LET procedure, conducted under regional anesthesia with the patient in supine position, the knee in 90 degrees. A thigh tourniquet was used with 250 mmHg pressure. An incision was made at the lateral aspect of the knee, from the lateral epicondyle towards Gerdy's tubercle. The iliotibial band was exposed and a 9 by 1 cm strip was excised from the middle part of the iliotibial band, leaving its distal end attached to Gerdy's tubercle (Fig. 5). The free end of the graft was whipstitched with a high strength braided suture (Vicryl 2, Ethicon). Then the graft was rerouted by a curved clamp deep to the lateral collateral ligament. A femoral tunnel was created just proximal and anterior to the lateral epicondyle by drilling a 2.4 mm eyelet pin through the lateral cortex out of the medial femoral cortex in slightly anterior and proximal direction. The guidepin was overreamed by a 6 mm cannulated reamer to a minimum depth of 30 mm. Then the passing suture was threaded through the eyelet and pulled into the femoral tunnel. The graft was fixated in 30 degrees of knee flexion with a 7 mm interference screw, keeping the foot in neutral tibial rotation under slight tension over the graft (20N). The iliotibial band, subcutaneous tissue, and skin were closed layer by layer using absorbable sutures.

Postoperatively, patients were mobilized with two crutches and weightbearing was allowed as tolerated for a minimum of two weeks. Free passive and active movement were allowed under physiotherapy supervision.

Pre- and postoperative outcome scores were assessed, including Lachman and Pivot Shift tests, Lysholm and Tegner scores. All four patients had an uneventful recovery after their LET procedure and showed improvement of outcome scores at one-year follow-up. There was improvement in Lachman test, Pivot test and Lysholm score post-operatively. Postoperative Lachman scores of all patients were + 1 that showed good anterior-posterior stability, while postoperative Pivot score decreased from +2 to negative for all patients, showing restored rotatory stability. All patients reported improved adequate subjective stability. Postoperative Lysholm scores were increased with a mean of 19.75 points, and Tegner scores returned to pre-injury level (Table 2).

**Table 2:** Pre- and postoperative outcome scores.

	Patient 1	Patient 2	Patient 3	Patient 4
Lachman pre op	1+	2+	1+	1+
Lachman post op (1 yr)	1+	1+	1+	1+
Pivot shift grade pre op	2	2	2	2
Pivot shift grade post op (1 yr)	Negative	Negative	Negative	Negative
Lysholm score preop	47	77	46	59
Lysholm score postop (1 yr)	93	83	61	71
Tegner score (1 yr)	4	4	4	4

Abbreviation: yr, year; op, operation.

## 3 Discussion

The four cases presented demonstrate an isolated LET to be a feasible treatment option for persistent rotatory instability after ACLR in patients with a moderate activity level. A rationale for the additional effect of LET procedures can be found in studies that investigate the role of anterolateral structures of the knee. While a common perception has been that the ACL is paramount for rotational control of the knee (additional to its role in controlling anterior translation of the knee), recent biomechanical studies have shown that the anterolateral structures are in fact the primary stabilizers of internal rotation [10]. Concurrently, clinical findings – at both the time of surgery and during radiological examinations – indicate that these anterolateral structures are commonly injured when an ACL tear is present [11].

The modified Lemaire is a LET procedure that is indicated as a supplement to ACLR in selected cases. In this procedure a transposed graft from the iliotibial band is tunneled deep to the lateral collateral ligament, with only the need for a single incision requiring no additional tendon harvesting. It is easy to perform with the bony landmarks for graft insertion being identifiable through the open approach used, which is posterior and proximal to the lateral femoral epicondyle and the origin of the LCL. By using only one fixation device at the femoral insertion, there is a reduced risk of graft slippage as well as reduced costs related to the procedure [3].

Addition of the LET procedure to address persistent anterolateral rotatory laxity has been performed in combination with ACLR procedures [3], [10]. To date, there are no publications about isolated secondary LET (performed as a modified Lemaire) after primary ACLR with residual rotatory instability.

Our four patients where successfully treated with an isolated secondary LET procedure (modified Lemaire) for residual anterolateral rotatory instability after primary ACLR. These results are supported by Hewison et al. [8], who described that the addition of a LET to ACLR led to improved knee stability, decreased rotational knee laxity and a decreased rate of ACL failure. Helito et al. [12] described one case with residual rotatory instability after ACL reconstruction, successfully treated with a secondary ALL reconstruction using an allograft semitendinosus. Although the clinical evidence is currently lacking to support the clear indications for lateral extraarticular procedures as a primary augmentation to ACL reconstruction, there are likely to be individuals who may still benefit from this procedure, and the ALC Consensus Group Meeting (2017) gave a recommendation for this procedure in selected cases, those are 1) in revision cases, 2) high-grade pivot shift, 3) generalized ligamentous laxity/ genu recurvatum, and 4)young patients returning to pivoting activities [10].

A long-standing fear with the LET has been the perceived risk of lateral compartment osteoarthritis associated with such procedures [13]. A suggested mechanism for the development of lateral compartment osteoarthritis is over-constraining of the lateral compartment, leading to an increase in tibiofemoral cartilage contact pressures. Although there is no clear evidence that supports such a link, this has been a prevailing opinion among knee surgeons. In a cadaveric study investigating the effect of graft tensioning protocols on intra- articular tibiofemoral contact pressures, a low risk of increased intra-articular contact pressures was observed [14]. The key factors for keeping intra-articular pressures normal included: (1) keeping the knee in a neutral rotation, and (2) using only a moderate graft tension (20 N) when fixating the anterolateral graft. These findings highlight important technical factors to be considered in the anterolateral procedures [15].

Two of our cases appeared to have a steep orientation of the graft. This is a well-known consequence of the transtibial drilling technique, used in these patients. Although a steep orientation of the graft would hypothetically lead to increased rotatory instability, clinical results of the transtibial technique versus the anteromedial portal technique are not that different [16]. Another point to address is the fact that all four described cases were female patients. We believe female patients are more likely to develop residual rotatory instability after ACL reconstruction. There is evidence demonstrating an increased rotational laxity in females, which also seems one of the reasons why they are more prone to ACL injuries than males [17], [18].

## 4 Conclusions

Adding a lateral extra-articular tenodesis can improve patient outcome without the need for a complete ACL revision and its obligatory intense postoperative rehabilitation, in moderate active patients presenting with residual rotatory instability after ACL reconstruction.

## Sources of funding

This research did not receive any specific grant from funding agencies in the public, commercial, or not-for-profit sectors.

## Ethical approval

N/a.

## Consent

Informed consent was obtained from the patients.

## Research registration

N/a.

## Guarantor

HvdV (2nd author) is a guarantor of this study.

## Provenance and peer review

Not commissioned, externally peer-reviewed.

## CRediT authorship contribution statement

RD contributed in study concept or design, data analysis or interpretation, and writing the paper.

HvdV contributed in study concept or design, data collection, data analysis or interpretation, revising the paper.

## Declaration of competing interest

None declared.
